# Field Trials Need Genetic Localization, Not Geographic Isolation

**DOI:** 10.4269/ajtmh.25-0520a

**Published:** 2025-12-04

**Authors:** Kevin M. Esvelt

**Affiliations:** Media Laboratory Massachusetts Institute of Technology Cambridge Massachusetts, USA E-mail: esvelt@mit.edu

Dear Editor,

In the article, *“Is Gene Drive Research Losing Traction?”* Lanzaro and Kormos correctly identify a travesty: years of remarkable technical progress in gene drive development have yielded zero field trials.^1^ With hundreds of thousands of children dying annually, a single day of unnecessary delay is a moral catastrophe. However, their proposed solution – releasing gene drives on “ecological islands” – reveals a fundamental problem: “gene drive” has become dangerously imprecise terminology.

There’s a vast difference between a self-propagating gene drive construct designed to spread from a single individual to an entire species, and a localized construct like a split or daisy drive that becomes ten-fold or a hundred-fold more common in the population near the release site, then diminishes. By conflating these radically different technologies – and apparently placing their faith in geographic containment for what any reader would assume to be self-propagating systems – the authors risk unplanned continent-wide dispersal. Escape and international spread from what was supposed to be a confined field trial would rightfully damage public trust, likely precluding the follow-up releases necessary to permanently eradicate malaria, New World screwworm, and eventually schistosomiasis. Yes, there’s a possibility that a sudden collapse in malaria cases would occur, and be welcomed, but with this escape the scientific and regulatory communities would be revealed as incompetent, and the public response might depend on who illegally moved the organisms and why – a risk we can ill afford.

More generally, the conflation of self-propagating and localized gene drive systems is paralyzing progress. Self-propagating gene drives will ultimately affect entire continents – everyone living alongside the target species, everywhere it exists. Understandably, ethicists and civil society organizations demand input from all potentially affected populations. But consulting millions of people across sub-Saharan Africa isn’t feasible, creating an impossible standard that blocks all progress, precisely because it is also misapplied to localized interventions.

The solution lies in highlighting the distinction and pressing forward with localized systems that lose their fitness advantage over generations but otherwise introduce exactly the same intervention as their more powerful relatives. Models and experiments in disease-carrying mosquitoes have demonstrated that the effects of localized systems remain confined to a limited area.^2^^,^^3^ This controllability is precisely what makes self-limiting systems suitable for field trials. Localized approaches require input only from communities in the trial region, because we can be confident that others will not be affected. My laboratory’s work towards engineering wild mouse populations on Nantucket and Martha’s Vineyard has demonstrated that pioneering local non-scientist communities are fully capable of guiding research and the development of applications intended to exclusively change their environment.^4^

In contrast, releasing a self-propagating system expected to confer a fitness advantage isn’t a trial – it’s deployment. Geographic barriers cannot contain gene drives. The authors cite “ecological islands within the mainland,” but *Anopheles* populations maintain gene flow across seemingly isolated habitats. More critically, experimenters cannot control this gene flow. The smuggling of rabbits with hemorrhagic disease virus from Australia to New Zealand demonstrates that motivated individuals will transport organisms across even the strictest biosecurity barriers.^5^ A single gravid mosquito in a vehicle can breach any geographic “containment,” never mind a determined individual with a bag.

Once a self-propagating drive is released, the only robustly demonstrated recall mechanism is an immunizing reversal drive overwriting the initial changes.^6^ That might, at best, preserve ecological safety, but not public trust. Would you trust scientists whose “field trial” spreads beyond the intended region and ultimately edits the bulk of an entire species?

The path forward is clear: advance genuinely self-limiting systems with local community support. Localized systems can demonstrate efficacy and ecological effects across multiple environments while maintaining experimental control. Success would build the empirical foundation and public trust necessary for the later approval of self-propagating drive releases.

The authors’ frustration is understandable – I share it. But suggesting that we can control gene flow through geography alone, if that is indeed what their Perspective asserts, isn’t bold. It’s reckless. If one death in a poorly planned clinical trial set gene therapy back a decade, the unauthorized editing of an entire species would vindicate every critic and delay the gene drive field for even longer.^7^ We cannot afford that delay.

Let’s run the trials we should have conducted years ago – with systems designed to stay localized, guided by the communities that choose to host them.
